# Attenuation of inflammatory responses by (+)-syringaresinol via MAP-Kinase-mediated suppression of NF-κB signaling *in vitro* and *in vivo*

**DOI:** 10.1038/s41598-018-27585-w

**Published:** 2018-06-15

**Authors:** Vivek K. Bajpai, Md Badrul Alam, Khong Trong Quan, Mi-Kyoung Ju, Rajib Majumder, Shruti Shukla, Yun Suk Huh, MinKyun Na, Sang Han Lee, Young-Kyu Han

**Affiliations:** 1 Department of Energy and Materials Engineering, Dongguk University-Seoul, Seoul, 04620 Republic of Korea; 2 Departments of Food Science and Biotechnology, Graduate School, Kyungpook National University, Daegu, 41566 Republic of Korea; 3 College of Pharmacy, Chungnam National University, Daejeon, 34134 Republic of Korea; 4 Department of Biological Sciences, Macquarie University, Sydney, NSW 2109 Australia; 5 NSW Department of Primary Industries, Elizabeth Macarthur Agricultural Institute (EMAI), Menangle, NSW 2567 Australia; 6 Department of Biological Engineering, Inha University, 100 Inha-ro, Nam-gu, Incheon, 22212 Republic of Korea

## Abstract

We examined the anti-inflammatory effects of (+)-syringaresinol (SGRS), a lignan isolated from *Rubia philippinensis*, in lipopolysaccharide (LPS)-stimulated RAW 264.7 cells using enzyme-based immuno assay, Western blotting, and RT-PCR analyses. Additionally, *in vivo* effects of SGRS in the acute inflammatory state were examined by using the carrageenan-induced hind paw edema assay in experimental mice. As a result, treatment with SGRS (25, 50, and 100 μM) inhibited protein expression of lipopolysaccharide-stimulated inducible nitric oxide synthase (iNOS), cyclooxygenase-2 (COX-2), and nuclear factor kappa B (NF-κB) as well as production of nitric oxide (NO), prostaglandin E2 (PGE2), tumor necrosis factor-alpha (TNF-α), interleukin-1beta (IL-1β), and interleukin-6 (IL-6) induced by LPS. Moreover, SGRS also reduced LPS-induced mRNA expression levels of iNOS and COX-2, including NO, PGE2, TNF-α, IL-1β, and IL-6 cytokines in a dose-dependent fashion. Furthermore, carrageenan-induced paw edema assay validated the *in vivo* anti-edema effect of SGRS. Interestingly, SGRS (30 mg/kg) suppressed carrageenan-induced elevation of iNOS, COX-2, TNF-α, IL-1β, and IL-6 mRNA levels as well as COX-2 and NF-κB protein levels, suggesting SGRS may possess anti-inflammatory activities.

## Introduction

The inflammatory response, a physiological reaction to infection or damage, plays a vital role in the natural defense mechanisms of the body to maintain immune homeostasis^1^. Inflammation refers to the body’s normal protective response to tissue injury caused by physical trauma, toxic chemicals or microbiological agents, and the classical signs of inflammation are skin redness, swelling, pain, heat, and loss of function^2^. The process of inflammation involves changes in blood flow, destruction of tissues, increased vascular permeability and the synthesis of pro-inflammatory mediators. The injured cells and blood proteins are the sources of inflammatory mediators. The inflammatory process is defined in two critical phases, one as an acute phase, where inflammation occurs a few minutes after tissue damage while failure to acute phase results in the chronic inflammation^2^. Nevertheless, instability of immune homeostasis as well as a prolonged inflammatory response can result in the development of various chronic diseases such as autoimmune disorders, cancer, and vascular diseases^3^. Phagocytosis of pathogens via toll-like receptors (TLRs), which distinguish molecular patterns of lipopolysaccharide (LPS), a pathogen-derived material, results in the activation of immune cells (macrophages, neutrophils, and dendritic cells), thereby triggering inflammatory responses^4^. These immune cells upon activation mediate enhanced production of various pro-inflammatory proteins/enzymes that include cyclooxygenase-2 (COX-2) and inducible nitric oxide synthase (iNOS) along with production of other pro-inflammatory cytokines, such as interleukins (IL-1β and IL-6) and tumor necrosis factor-α (TNF-α). These pro-inflammatory biomarkers are known as important mediators of inflammatory responses^5^. Activation of nuclear factor-kappa B (NF-κB) plays a significant role in the regulation of protein expression levels of iNOS and COX-2, which eventually produce nitric oxide (NO) and prostaglandin E2 (PGE2)^6,7^. NF-κB is involved in the trans-activation of a number of genes as an important transcriptional factor, which regulate both immune-inflammatory and acute-inflammatory responses, including the cell survival and tumorigenesis^8,9^. Furthermore, there is a great deal of involvement of cytokines such as tumor necrosis factor (TNF)-α, interferon (IFN)-γ and interleukin (IL)-6 in the development of diseases associated with inflammation and inflammatory responses^10^.

Mitogen-activated protein kinases (MAPKs) such as extracellular signal-regulated kinase (ERK), p38 mitogen-activated protein kinase (p38 MAPK), and c-Jun NH2-terminal kinase (JNK), comprising a group of signaling pathways, play vital roles in the regulation of cell differentiation and growth, and their phosphorylation is known to be a critical component in the production of NO and pro-inflammatory cytokines in activated macrophages^9,11^. Thus, mounting research has focused on identifying safe candidate materials with a preventive ability to treat inflammatory diseases through their diverse inhibitory action against upstream signaling events involved in the expression profile of inflammatory genes.

*Rubia philippinensis* is a rambling and a low climbing perennial herb that grows in the Southern part of Vietnam. Local communities have long utilized this medicinal plant to treat ordinary ailments such as wounds, inflammation, and skin infections^12^. Previous investigations of the species have resulted in the purification of arborinane triterpenoids, which show promising effects on the prevention and treatment of atherosclerosis^13^. Additionally, rubiarbonone C, a popular chemical entity isolated from *R. philippinensis*, has been shown to inhibit abnormal proliferation and migration of vascular smooth muscle cells, which plays an important role in the pathophysiology of atherosclerosis. The mechanism by which rubiarbonone C regulates vascular remodeling was further clarified through focal adhesion kinase (FAK), MAPK, and STAT3 Tyr705^14^. In searching for bioactive components from *R. philippinensis*, in this study, (+)-syringaresinol was also isolated as a major compound. In addition, Cai *et al*.^15^ also reported isolation and characterization of (+)-syringaresinol from *Acanthopanax koreanum* along with some other phytoconstituents, including eleutheroside E, tortoside A, and hemlarlensin which were enough cable to inhibit a cytoplasmic protein NFAT playing a significant role in the induction of immune responses.

There is a great increasing demand of natural products as herbal medicines due to their being less toxic, affordable, easily available, and with fewer adversary effects on human. As a part of prior research examining the biological potential of effective phytochemicals and to minimize the side effects of commercial anti-inflammatory drugs, such as non-steroidal anti-inflammatory drugs (NSAIDs), a lignan, (+)-syringaresinol (SGRS) isolated in this study from *R. philippinensis* was assessed for its potent anti-inflammatory effects both *in vitro* and *in vivo*. In addition, relevant targets involved in the regulation of inflammatory responses were studied to estimate the precise anti-inflammatory mode of action of SGRS. Current research focused on the evaluation of detailed anti-inflammatory mechanism of SGRS in terms of its effect on LPS-stimulated macrophages that influence MAPK signaling pathways. The findings demonstrate that SGRS attenuated the inflammatory response via down-regulation of NF-κB by activating p38 and JNK proteins in RAW 264.7 cells.

## Materials and Methods

### Plant materials

Root samples of *Rubia philippinensis* were claimed from the National Park of Lam Dong city, Vietnam. Taxonomic identification of root samples was done at the National Institute of Medicinal Materials, Vietnam by Dr. Phuong Thien Thuong. Specimen sample of *R. philippinensis* root was deposited under the herbarium identification number VDL20140801 at the National Institute of Medicinal Materials, Hanoi, Vietnam. Also, the same root sample was deposited under same specimen identification number at the Pharmacy College of Chungnam National University, Daejeon, Korea.

### Extraction, isolation, and characterization of (+)-syringaresinol (SGRS)

(+)-Syringaresinol was acquired from the root samples of *R. philippinensis* using various chromatographic techniques. In brief, ethanol-mediated root extract of *R. philippinensis* (150 g) was suspended in H_2_O (1.5 L) followed by subsequent partitioning using CH_2_Cl_2_ (2 L × 3) to yield CH_2_Cl_2_ extract. The CH_2_Cl_2_-soluble fraction (50 g) was subjected to silica gel VLC and eluted with *n*-hexane-EtOAc (20:1, 10:1, 5:1, 3:1, 2:1) and CHCl_3_-MeOH (8:1) to afford six fractions (D-1 → D-6). Fraction D-6 (10 g) was divided into 11 sub-fractions (D-6-1 → D-6-11) using MPLC with a gradient of MeOH-H_2_O (10:90 → 100:0, 7 L). (+)-Syringaresinol (*t*_R_ 42.0 min, 70 mg) was obtained from D-6-3 (360 mg) by eluting HPLC with MeOH-H_2_O (45:55, 4 mL/min, UV 254 nm).

(+)-Syringaresinol (SGRS): brownish amorphous powder, ${[\alpha ]}_{{\rm{D}}}^{25}$+8 (*c* 0.1, CHCl_3_), ^1^H NMR (300 MHz, methanol-*d*_4_): 6.69 (4 H, s, H-2′, H-2″, H-6′, H-6″), 4.74 (2 H, d, *J* = 4.0 Hz, H-2, H-6), 4.29 (2 H, dd, *J* = 6.6, 9.0 Hz, H-4a, H-8a), 3.91 (2 H, dd, *J* = 3.1, 9.0 Hz, H-4b, H-8b), 3.87 (12 H, s, OCH_3_-3′, 3″, 5′, 5″), 3.16 (2 H, m, H-1, H-5). ^13^C NMR (75 MHz, methanol-*d*_4_) 55.4 (C-1, C-5), 87.5 (C-2, C-6), 72.7 (C-4, C-8), 133.1 (C-1′, C-1″), 104.4 (C-2′, C-2″, C-6′, C-6″), 149.2 (C-3′, C-3″, C-5′, C-5″), 136.1 (C-4′, C-4″), 56.7 (OCH_3_-3′, 3″, 5′, 5″).

### Cell culture and chemicals

RAW 264.7 cells were cultured in Dulbecco’s Modified Essential Medium (DMEM) supplemented with 100 units/mL of penicillin-streptomycin and 10% heat-inactivated fetal bovine serum (FBS) (Grand Island, NY, USA) under a humidified 5% CO_2_ atmosphere at 37 °C. 3-(4,5-dimethylthiazol-2-yl)-2,5-diphenyltetrazolium bromide (MTT), 2′,7′-dichlorofluorescin diacetate (DCFH-DA), phosphate-buffered saline (PBS, pH 7.4), and dimethylsulfoxide (DMSO) were procured commercially (Sigma Aldrich, St. Louis, MO, USA).

### Cell viability and nitric oxide determination

The MTT assay was employed to evaluate the cell viability of SGRS. In brief, a 24-well plate was used to palate the RAW 264.7 cells at the density of 1 × 10^5^ per well followed by incubation at 37 °C for 24 h. For the treatment of the cells, various concentrations (25, 50, and 100 μM) of SGRS were used for 2 h before LPS (1 μg/mL) stimulation, including a vehicle control followed by incubation for an additional 18 h at 37 °C. Afterwards, aliquots (100 μL) of the cell-free culture medium were taken for the measurement of NO following the Griess reaction method. For the determination of cell viability, a previously reported method was adopted^16^.

### TNF-α, IL-1β, IL-6, and PGE_2_ assays

RAW 264.7 cells (5 × 10^5^ cells/mL) were incubated in media for 16 h. Cells were then pre-treated with various concentrations (25, 50, and 100 μM) of SGRS for 1 h, followed by LPS (1 μg/mL)-mediated stimulation. The cell culture supernatant was collected from the cells after 20 h of LPS-mediated cell stimulation, and an enzyme-linked immunosorbent assay (ELISA) was performed for the quantitative determination of the levels of TNF-α, IL-1β, IL-6, and PGE2.

### NF-kB reporter assay

The pNF-κB-luc plasmid (Beyotime Biotechnology, Nantong, China) containing four NF-κB-binding motifs (GGGAATTTCC) and pRL-SV40 (Renilla luciferase driven by SV40) reporter constructs (Promega, Madison, WI, USA) were complexed using GenJetTM Plus DNA *In Vitro* Transfection Reagent (Signagen, Maryland, USA) in 100 mL of serum-free culture medium. Then, RAW 264.7 cells seeded in six-well plates (Corning, New York, NY, USA) were transfected by adding the mixture. Luciferase activity was assayed after cells were treated with LPS or various concentrations of SGRS using the Dual-Luciferase^®^ Reporter Assay System (Promega, Madison, WI, USA).

### Preparation of nuclear extracts

After dishes were washed with ice-cold PBS, cells were scraped and transferred to microtubes. Cells were swollen by adding lysis buffer [10 mM HEPES (pH 7.9), 10 mM KCl, 1.5 mM MgCl_2_, 1 mM dithiothreitol, 0.2% NP-40, and protease inhibitor cocktail (Roche Diagnostics, Indianapolis, IN, USA)] and then incubated for 10 min on ice and centrifuged 15,000 × g for 5 min at 4 °C. Pellets containing crude nuclei were re-suspended in 50 μL of extraction buffer (20 mM HEPES (pH 7.9), 1.5 mM MgCl_2_, 1 mM dithiothreitol, 420 mM NaCl, 20% glycerol, and protease inhibitor cocktail), incubated for 30 min on a shaker at 4 °C, and centrifuged at 16,000 × g for 10 min in order to obtain supernatants (nuclear extracts).

### Carrageenan-induced paw edema

Eight-week-old ICR mice were obtained from Central Lab Animals, Inc. (Seoul, Korea) and housed in an air-conditioned animal room at a temperature of 23 ± 1 °C, humidity of 55 ± 5%, and 12 h/12 h light/dark cycle with ad libitum access to water and standard laboratory diet. The animals were acclimatized for 1 week and randomly divided into five groups of five mice each. The experiment was conducted in accordance with the guidelines for animal experiments issued by the Kyungpook National University and approved by the Institutional Animal Care and Use Committee of Kyungpook National University (KNU-2017-0035). Mice (N = 25) were randomly divided into four groups (five animals/group): treatment naïve control group (group-1), CA control group (group-2), indomethacin group (group-3), and 50 mg/kg/day SGRS group (group-4). For oral administration, SGRS (dissolved in 40% polyethylene glycol) was administered at the dose of 50 mg/kg/day for 4 consecutive days. For positive control, standard anti-inflammatory drug, indomethacin was employed. For induction of acute phase inflammation, a subcutaneous injection of carrageenan (1%) was administered (60 μL per animal) into the right hind paws of mice after 1 h SGRS or vehicle treatment. A plethysmometer was used for measuring the paw volumes hourly for 4 h after carrageenan injection, after which mice were euthanized. The right hind paw skin was then expunged and immediately frozen in a nitrogen tank for RT-PCR and Western blotting analyses.

### mRNA analysis by semi-quantitative RT-PCR

To evaluate mRNA expression levels, RAW 264.7 cells were pre-treated with SGRS (25, 50, and 100 μM) for 30 min before incubation with LPS (1 μg/mL) for 6 h. Total RNA was isolated with TRIzol Reagent (Invitrogen Co., Carlsbad, CA, USA) according to the manufacturer’s instructions. Semi-quantitative RT-PCR reactions were conducted as previously reported with minor modifications^17^. In brief, to prepare a cDNA pool from RNAs, total RNA (2 μg) was transcribed using an RT-&GO Mastermix (MP Biomedicals, Seoul, Korea), and the product was used as the PCR template. Reverse transcription PCR (RT-PCR) was performed using a PCR Thermal Cycler Dice TP600 (TAKARA Bio Inc., Otsu, Japan) using the specific primer sequences. Information on specific oligonucleotide primers used in this study for mouse transcripts is given in Table S1. For the visualization of PCR products, ethidium bromide staining was preformed following electrophoresis. An Image Lab™ Software (version 5.2.1) was used for analyzing the bands.

### Western blot analysis

Macrophage RAW 264.7 cells were pretreated using above-mentioned concentrations of SGRS or vehicle for 2 h followed by stimulation with LPS (1 μg/mL) for 6 h. Primary and secondary antibodies were obtained commercially (Santa Cruz Biotechnology, Cruz, CA, USA). Ten micrograms of total proteins were separated by SDS-PAGE. Proteins were electro-transferred to nitrocellulose membranes after electrophoresis, blocked with 5% non-fat milk in TBST buffer, and blotted with each primary antibody (1:1000) and with corresponding secondary antibody (1:5000). The antigen-antibody reaction was detected using an ECL solution system (Perkin Elmer). An Image Lab™ Software (version 5.2.1) was used for analyzing the bands. The membrane was stripped by using stripping buffer (Restore^TM^ Western Blot Stripping Buffer, Thermo Scientific, Rockford, IL, USA) for the screening of various biomarkers. Briefly, the membrane was immersed completely in the stripping buffer for 15 min in shaking motion. The stripping buffer was then carefully, but thoroughly washed with TBST twice for 10 min. Then the membrane was again blocked with 5% non-fat milk in TBST buffer, and blotted with β-actin primary antibody (1:1000) and with corresponding secondary antibody (1:5000). The antigen-antibody reaction was detected using an ECL solution system (Perkin Elmer). An Image Lab™ Software (version 5.2.1) was used for analyzing the bands.

### Statistical analysis

Data were presented as the mean ± SD followed by one-way ANOVA analysis. The value (p < 0.05) was considered significant for the differences. For all data analyses, Window’s SPSS Software (Version 10.07 (SPSS, Chicago, IL, USA) was used.

## Results

### Identification and characterization of (+)-syringaresinol (SGRS)

**Figure 1:**
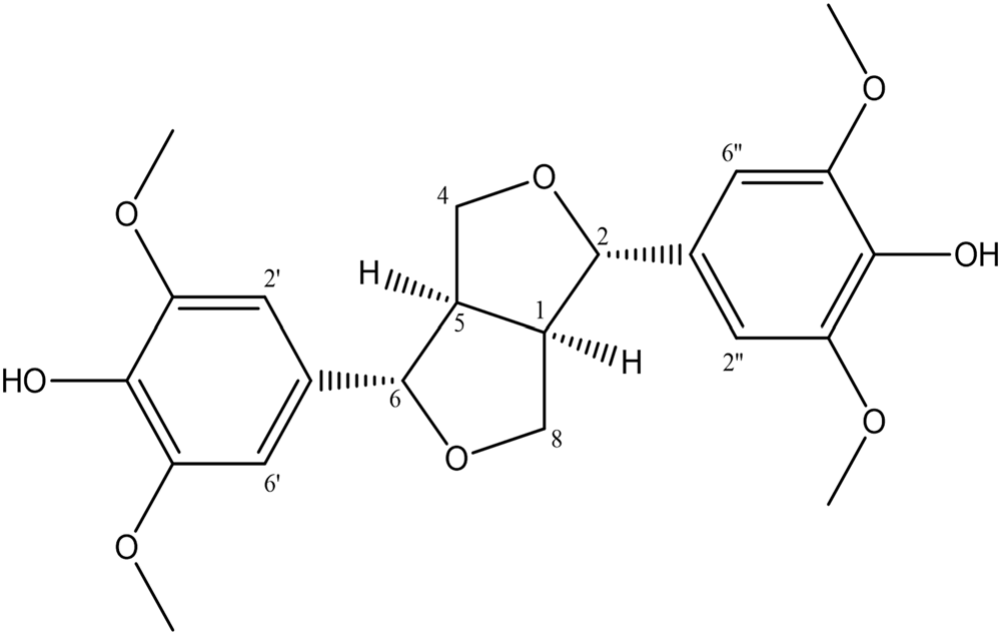
Chemical structure of (+)-syringaresinol, a lignan isolated from *Rubia philippinensis*.

The ^1^H NMR data of the purified compound, with a specific rotation value ${[\alpha ]}_{D}^{25}+8$ (*c* 0.1, CHCl_3_)^18,19^, displayed four symmetric protons at *δ*_H_ 6.69 (4 H, brs, H-2′, H-6′, H-2″, H-6″). In addition, two oxygenated methines at *δ*_H_ 4.74 (2 H, d, *J* = 4.0, H-2, H-6), two oxygenated methylene functionalities at *δ*_H_ 4.29 (2 H, dd, *J* = 6.6, 9.0, H-4a, H-8a) and *δ*_H_ 3.91 (2 H, dd, *J* = 3.1, 9.0, H-4b, H-8b), and two methines at *δ*_H_ 3.16 (2 H, m, H-1, H-5), indicating two symmetrical tetrahydrofuran units, were observed in the ^1^H NMR data as well (Fig. S1). The ^13^C NMR spectrum (Fig. S2) showed a total of 18 signals for typical lignan derivatives along with four aromatic methoxy groups (*δ*_C_ 56.7). Based on the analysis of NMR spectroscopic data and specific rotation values, the compound was identified as (+)-syringaresinol (Fig. 1)^15^.

### Effect of SGRS on production of inflammatory mediators in LPS-induced RAW264.7 cells

**Figure 2:**
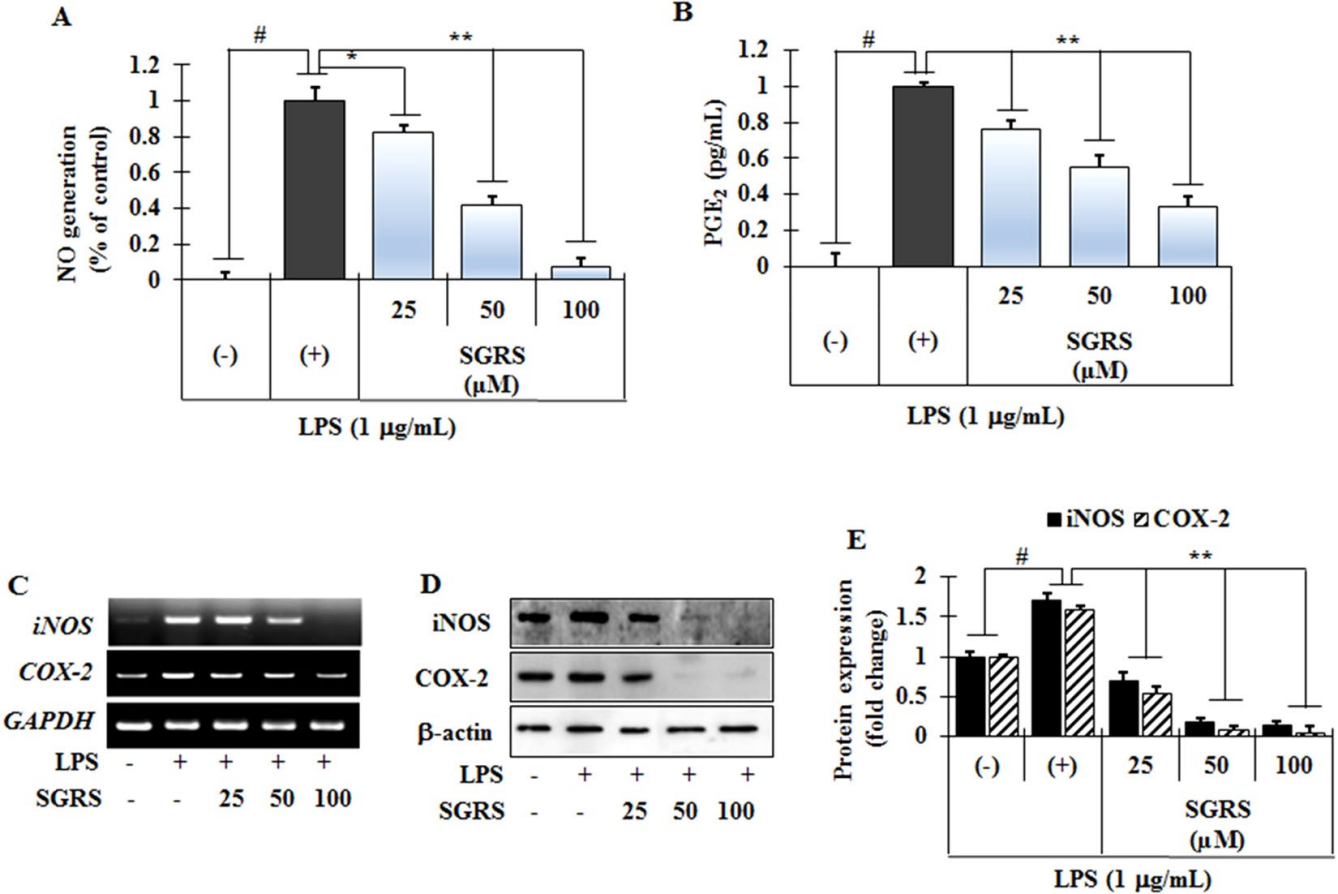
Inhibitory effects of (+)-syringaresinol (SGRS) on LPS-induced productions of (**A**) NO, (**B**) PGE_2_, (**C**) mRNA expression and (**D**) protein expression of iNOS and COX-2, respectively, (**E**) quantification of the protein expression of iNOS and COX-2 in RAW 264.7 cells. Cells (5 × 10^5^ cells/mL) were treated with various concentrations (25, 50, or 100 μM) of SGRS for 1 h, and then incubated with LPS (1 μg/mL). The membrane was subjected to stripping and re-probed for COX-2 and β-actin. Results are the mean ± S.D. of three separate experiments. ^#^p < 0.01 significant compared with vehicle-treated control; *p < 0.05 and **p < 0.01 significant compared with LPS alone.

Different dosages of SGRS (25, 50, and 100 μM) were used to evaluate its inhibitory effects on LPS-induced production of NO and PGE_2_ in RAW 264.7 cells. Compared to untreated control cells (column 1 of Fig. 2A), treatment with LPS significantly increased production of NO (column 2 of Fig. 2A). However, treatment with SGRS significantly and dose-dependently reduced NO production (column 3–5 of Fig. 2A). In addition, we examined the effect of SGRS on LPS-induced production of PGE_2_ (Fig. 2B). Compared to control cells (column 1 of Fig. 1B), LPS caused an increase in PGE_2_ production (column 2 of Fig. 2B), whereas SGRS treatment significantly reduced PGE_2_ production (column 3–5 of Fig. 2B) in a concentration-dependent manner. In contrast, SGRS did not affect cell viability, as measured by MTT assay at concentrations that inhibited the LPS-induced inflammatory response (Fig. S3). These results indicate that SGRS inhibits the LPS-induced inflammatory response without affecting cell viability.

To investigate whether or not the inhibitory effect of SGRS on NO and PGE2 production was due to inhibition of corresponding gene expression, mRNA and protein expression levels of inducible nitric oxide synthase (iNOS) and cyclooxygenase-2 (COX-2) were evaluated by RT-PCR and Western blot assays. As shown in Fig. 2C,D, LPS treatment markedly augmented transcription and translation levels of iNOS and COX-2, whereas cells pretreated with the indicated concentration of SGRS significantly attenuated LPS-induced iNOS and COX-2 gene and protein levels in a concentration-dependent manner (Fig. 2E). These data suggest that SGRS acts principally by suppressing NO and PGE_2_ production through regulation of gene transcription in activated macrophages.

### Effect of SGRS on the production of pro-inflammatory cytokines in LPS-induced RAW264.7 cells

**Figure 3:**
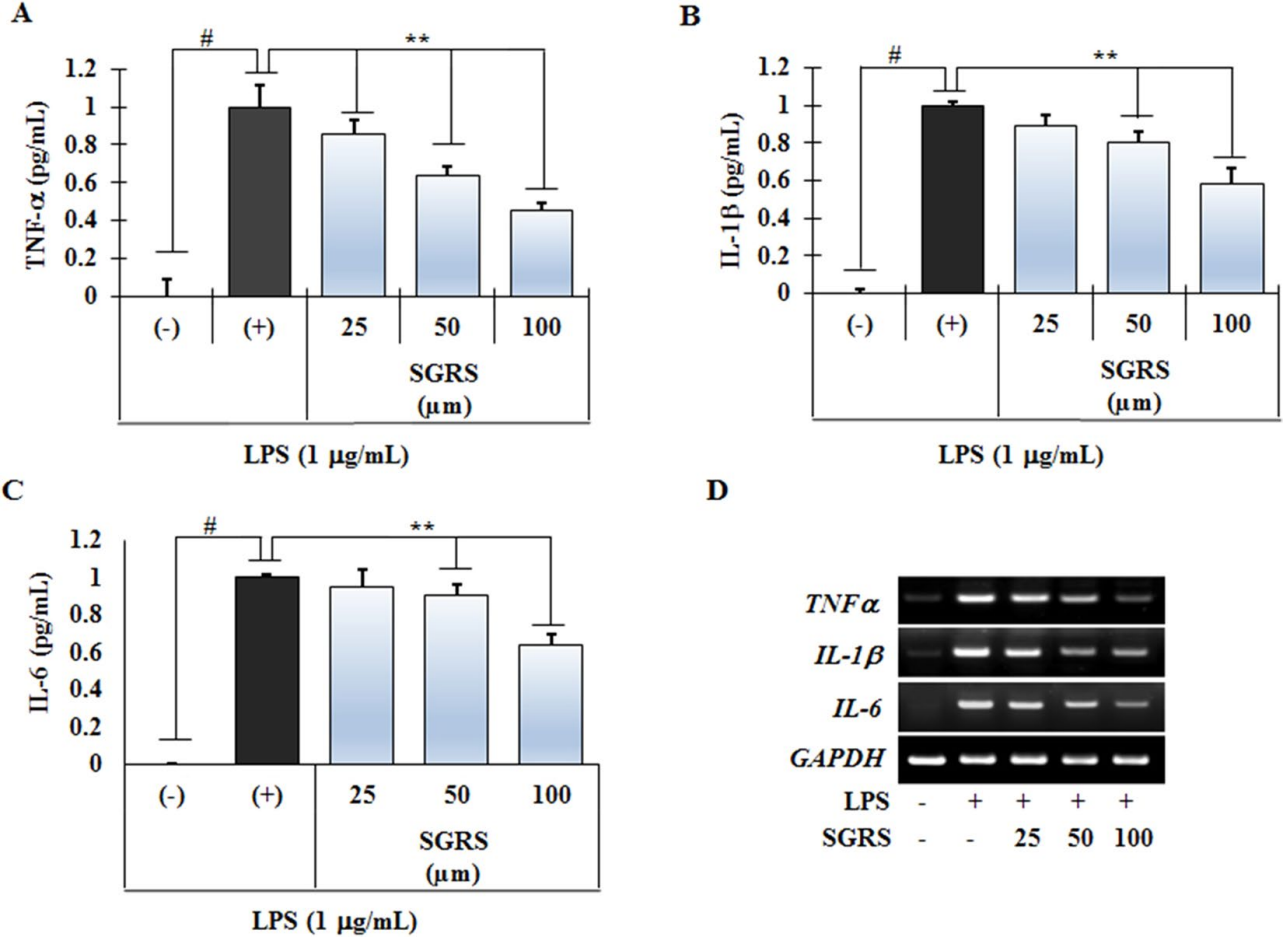
Inhibitory effects of (+)-syringaresinol (SGRS) on LPS-induced productions of (**A**) TNF-α, (**B**) IL-1β, (**C**) IL-6 and (**D**) mRNA expression of TNF-α, IL-1β, and IL-6 in RAW 264.7 cells. Cells (5 × 10^5^ cells/mL) were treated with various concentrations (25, 50, or 100 μM) of SGRS for 1 h, and then incubated with LPS (1 μg/mL) for 20 h. Results are the mean ± S.D. of three separate experiments. ^#^p < 0.01 significant compared with vehicle-treated control; *p < 0.05 and **p < 0.01 significant compared with LPS alone.

Next, we investigated whether or not SGRS inhibits production of the pro-inflammatory cytokines TNF-α, IL-1β, and IL-6 in LPS-stimulated RAW 264.7 cells by enzyme immunoassay. Compared with untreated controls (column 1 of Fig. 3A–C), LPS significantly increased production of TNF-α, IL-1β, and IL-6 in the culture supernatants of RAW 264.7 cells. However, treatment with SGRS significantly inhibited production of TNF-α, IL-1β, and IL-6 in a concentration-dependent manner (columns 3 to 5, Fig. 3A–C).

Since SGRS significantly inhibited LPS-induced production of pro-inflammatory cytokines (TNF-α, IL-1β, and IL-6), we performed RT-PCR to determine whether or not these inhibitory effects were related to changes at the mRNA level. As illustrated in Fig. 3D, mRNA levels of TNF-α, IL-1β, and IL-6 were markedly up-regulated in response to LPS, whereas treatment with SGRS inhibited mRNA expression in a dose-dependent manner. These results suggest that SGRS is effective in the inhibition of pro-inflammatory cytokine production via gene transcriptional regulation of TNF-α, IL-1β, and IL-6 in activated macrophages.

### Effect of SGRS on upstream signaling for NF-κB activation in LPS-induced RAW264.7 cells

**Figure 4:**
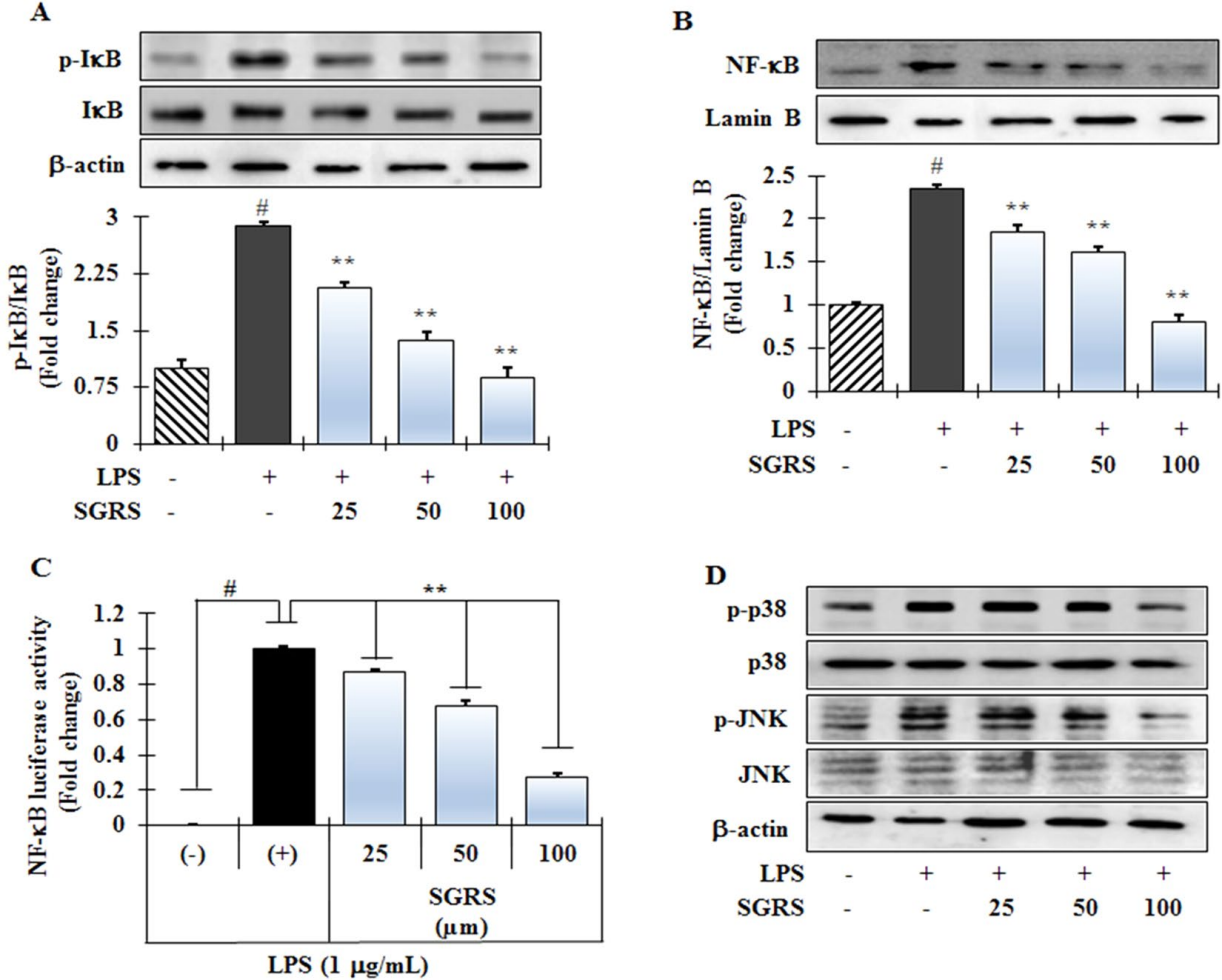
RAW 264.7 cells were treated with (+)-syringaresinol (SGRS) in the presence or absence of LPS (1 lg/ml). Effects of SGRS on (**A**) IκB phosphorylation (membrane was stripped and re-probed), and (**B**) NF-κB p65 subunit in LPS-stimulated RAW 264.7 cells by Western blotting. The quantification of relative band intensities from three independent experimental results was determined by densitometry. Results are the mean ± S.D. of three separate experiments. ^#^p < 0.01 significant compared with vehicle-treated control; *p < 0.05 and **p < 0.01 significant compared with LPS alone. (**C**) SGRS inhibits LPS-induced promoter activity of NF-κB in RAW 264.7 cells. The pNF-κB-luc containing four NF-κB binding motifs (GGGAATTTCC) and pRL-SV40 reporter constructs were transiently transfected into RAW 264.7 cells. Following different treatment, the promoter activity was detected using the Dual-Luciferase^®^ Reporter Assay System. (**D**) Effects of SGRS on MAPK phosphorylation in LPS-stimulated RAW 264.7 cells. The cellular proteins from the cells were used for the detection of phosphorylated or total forms of ERK1/2, p38, and JNK1/2 MAPKs.

This study also investigated whether or not SGRS has ability to block activation of the NF-κB pathway because regulation of inflammatory mediators in LPS-stimulated macrophages is transcriptionally implicated with the NF-κB. Phosphorylation of inhibitory kappa B (IκB) and its subsequent degradation by various stimuli is a critical step in NF-κB activation^20^, therefore, the effects of SGRS on LPS-induced degradation and phosphorylation of IκBα protein were investigated by immunoblotting analysis. SGRS inhibited LPS-induced phosphorylation of IκB in the cytosol in a dose-dependent manner (Fig. 4A). Free dimer activated subunits of NF-κB (p50/p65) can be translocated from the cytosol to the nucleus upon dissociation of IκB-α from NF-κB. Thus, in order to more specifically evaluate whether or not SGRS can affect the nuclear translocation of NF-κB, Western immunoblotting analysis for NF-κB was conducted using nuclear extracts of LPS-stimulated RAW 264.7 macrophages. Exposure of LPS alone significantly increased the amount of NF-κB in the nucleus (column 2, Fig. 4B). SGRS also inhibited LPS-induced nuclear translocation of NF-κB dose-dependently (column 3–5, Fig. 4B). An NF-κB-driven reporter construct in LPS-stimulated RAW 264.7 cells was employed, since in this system, luciferase reporter activity mediated by NF-κB is greatly induced^11^. Consistent with our previous data, NF-κB-driven luciferase activity in LPS-stimulated RAW264.7 cells was significantly reduced by SGRS in a dose-dependent manner (Fig. 4C), indicating that this extract blocked NF-κB activity. These findings indicate the potential role of NF-κB in the possible mode of action of SGRS in suppressing NO, PGE2, and pro-inflammatory cytokines in activated macrophages.

### SGRS attenuates MAPK phosphorylation in LPS-stimulated RAW264.7 cells

To confirm whether or not inhibition of NF-κB activation is mediated through MAPK pathways, we examined the effect of SGRS on LPS-stimulated phosphorylation of ERK1/2, JNK, and p38 MAPK in RAW264.7 cells. As depicted in Fig. 4D, LPS markedly induced phosphorylation of ERK1/2, JNK, and p38. Pre-treatment with SGRS significantly inhibited LPS-stimulated phosphorylation of p38 MAPK and JNK, whereas phosphorylation of ERK remained unchanged (data not shown). This result suggests that phosphorylation of p38 and JNK was inhibited by SGRS. However, the degree of inhibition was different for each MAPK, with the maximum inhibitory effect exerted on JNK. This result indicates that signal transduction by p38 and JNK might be effectively blocked by SGRS in activated macrophages.

### Inhibitory effects of SGRS on carrageenan-induced mouse hind paw edema

**Figure 5:**
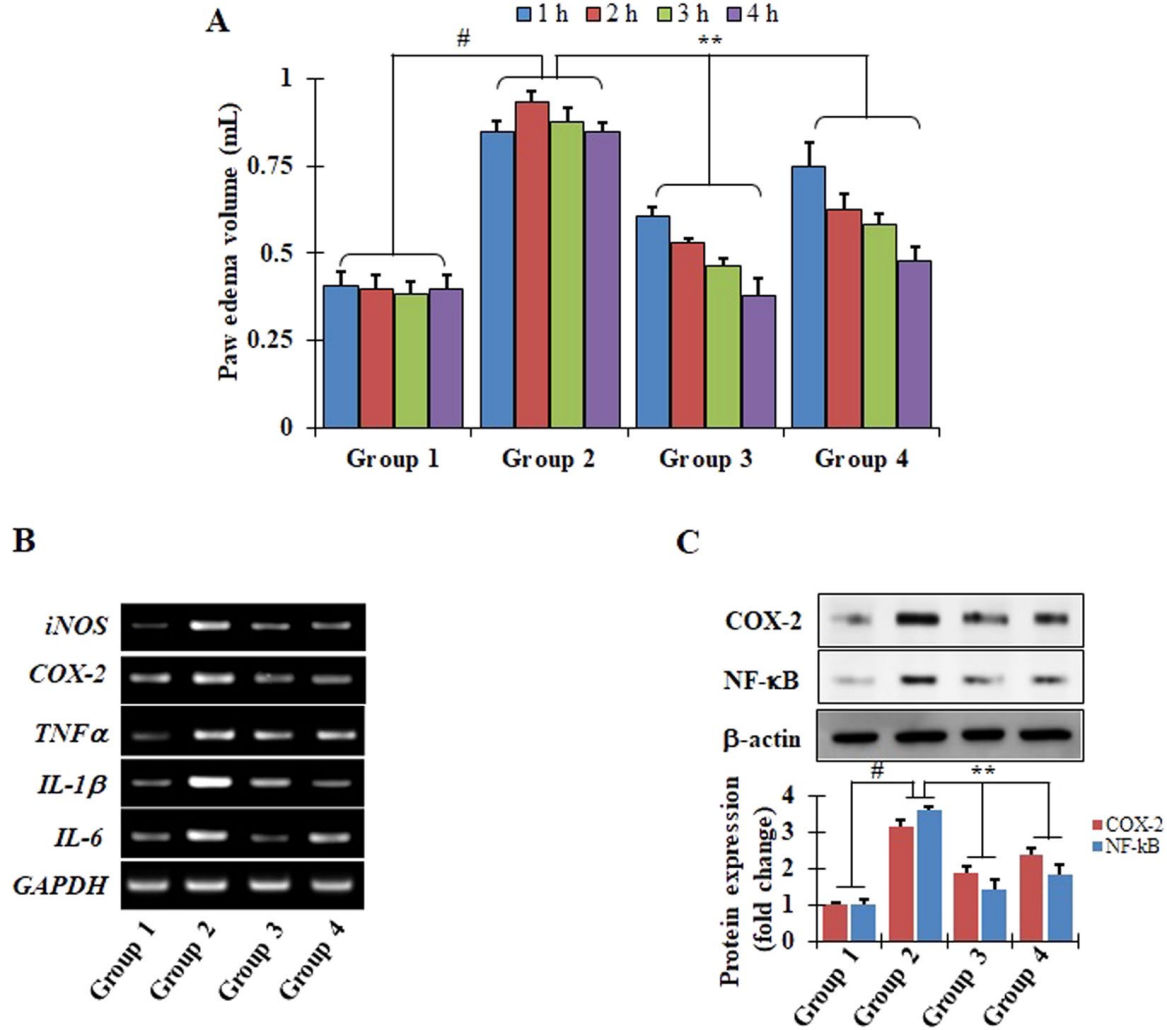
Inhibition of carrageenan (CA)-induced paw edema by (+)-syringaresinol (SGRS). (**A**) Paw volumes were measured 0–4 h after carrageenan injection as described in materials and methods. Results represent the mean ± S.D. of five animals. ^#^p < 0.01 significant compared with vehicle-treated control; *p < 0.05 and **p < 0.01 significant compared with LPS alone by one way ANOVA, followed by a post hoc Dunnet test. (**B**) mRNA expression of iNOS, COX-2, TNF-α, IL-1β, and IL-6 of CA-induced paw skin, (**C**) protein expression of COX-2, and NF-κB in CA-induced.

Treatment of mice with carrageenan resulted in significantly increased paw swelling in comparison with the control. However, pretreatment with indomethacin (10 mg/kg/day, p.o.), a positive control, significantly reduced paw edema formation. Similarly, treatment with SGRS (50 mg/kg/day, p.o.) significantly reduced paw edema volume (Fig. 5A). In addition, as expected, SGRS treatment significantly mitigated mRNA expression of inflammatory mediators (iNOS and COX-2) and various pro-inflammatory cytokines (TNF-α, IL-1β, and IL-6) compared to levels in the CA insult groups (Fig. 5B). Subsequently, protein expression of COX-2 and NF-κB was also suppressed in the SGRS-treated group compared to levels in the CA insult groups (Fig. 5C). These data suggest that SGRS attenuated carrageenan-induced inflammation in mice, likely via suppression of NF-κB signaling.

## Discussion

(+)-Syringaresinol (SGRS) is a naturally occurring lignan found in various plants, including flax seed (*Linum usitatissimum*), sesame seed (*Sesamum indicum*), Brassica vegetables, and grains (rye bran, wheat bran, oat bran, and barley bran)^21–23^. In this study, SGRS was isolated from the roots of *R. philippinensis* using various chromatographic techniques and characterized based on the spectral data analyses^15^. SGRS has also been characterized from the roots of *A. koreanum* with an ability to inhibit nuclear factor of activated T-cell protein^15^. Recently, SGRS has shown a significant potential to act as a neuromodulating agent by suppressing synaptic transmission via presynaptic transmitter release modulation^24^. Also, a furofuran-like lignan, syringaresinol-4-*O*-*β*-d-glucoside showed a potential efficacy in the treatment of lipid and glucose-based metabolic disorders^25^. Moreover, the role of SGRS has also been confirmed in the induction of mitochondrial biogenesis via activating PPARβ pathway in muscle cells^26^. However, the molecular mechanism responsible for the anti-inflammatory action of SGRS has not been evaluated so far. Hence, we evaluated anti-inflammatory effects of SGRS in LPS-induced RAW 264.7 cells as well as in a murine model of carrageenan-induced acute edematous inflammation in order to elucidate the relevant mechanism of action of SGRS.

Inflammation refers as a complex of biological responses to toxic stimuli during the process of host-defense^27^. Production of NO by NOS after carrageenan administration has significant involvement in the inflammation progression, whereas NO which is produced by iNOS is lately involved in maintaining the inflammatory responses^28^. More specifically, high levels of NO generated by inducible NO synthase (iNOS) have been defined as cytotoxic molecules in inflammation and endotoxemia^29^. Several studies investigated the anti-inflammatory effects of lignans from various plant sources on the production of NO/iNOS, PGE_2_/COX-2, TNF-α, and IL-1β in murine macrophages such as RAW 264.7 cells and observed down-regulation of inflammation-associated gene transcription^30–32^. In the present study, SGRS significantly inhibited LPS-induced production of NO and PGE_2_ in RAW 264.7 cells (Fig. 2A,B). Furthermore, SGRS attenuated LPS-induced gene expression as well as translation of iNOS and COX-2 (Fig. 2C,D). These findings indicate that NO inhibition and PGE_2_ production by SGRS might be due to the inhibition of iNOS and COX-2 up-regulation during macrophage activation by LPS.

TNF-α and IL-1β are pro-inflammatory cytokines involved in the pathogenesis of carrageenan-induced inflammation^33^. Also, IL-6 has been found to be interacted with a variety of target cells and associated with diverse immunological reactions^34^. In our study, we found that secretion (Fig. 3A–C) and mRNA expression of LPS-stimulated pro-inflammatory cytokines (Fig. 3D) was significantly inhibited by the treatment of SGRS. These findings indicate that inhibition of pro-inflammatory cytokines by SGRS may offer an ideal mean to treat inflammatory disorders.

Expression of iNOS, COX-2, and pro-inflammatory cytokines is regulated at the transcriptional level by NF-κB, which acts as their major transcriptional regulator^7^. In a resting cell, IκB-α retains NF-κB in the cytoplasm by masking nuclear localization sequences on NF-κB subunits^9^. Since IκB is dissociated from NF-κB upon phosphorylation, its content in the cytosol reflects the status of NF-κB, i.e. a higher IκB level indicates cytoplasmic localization of NF-κB while a higher p-IκB level indicates the nuclear localization of NF-κB. Many chemo-preventive and anti-inflammatory agents have been shown to reduce inflammatory symptoms by suppressing NF-κB expression. Our results show that treatment with LPS resulted in increased levels of NF-κB and p-I-κBα, whereas treatment with SGRS inhibited nuclear translocation of NF-κB (Fig. 4A) and levels of p-I-κBα (Fig. 4B). These findings suggest that treatment with SGRS inhibited NF-κB activation by suppressing the p-I-κBα level and nuclear translocation of NF-κB in LPS-induced RAW 264.7 cells. Taken together, the current study shows that inhibition of NF-κB activation by SGRS is associated with reduced induction of iNOS, COX-2, TNF-α, IL-1β, and IL-6.

It is well known that extracellular stimuli can activate the members of the MAPK family, such as serine and threonine kinases that have the ability to mediate cell surface signal transduction to the nucleus. The intracellular signals arising from MAPK cascades invariably lead to the activation of molecules that ultimately cause activation of NF-κB^35^. Hence, inhibition of any or all three MAPKs can be sufficient to block the inflammatory response. A number of lignans with anti-inflammatory properties have been reported to efficiently block LPS-induced phosphorylation of MAPKs^20,36^. However, the mode of action of lignans depends upon the substitution pattern in the core structure as well as the resultant derivatives that may target various proteins to bring about anti-inflammatory effects^37^. The present study demonstrated that SGRS significantly reduced the phosphorylation and degradation of IκB-α, thereby inhibiting translocation of NF-κB subunits from the cytosol into the nucleus^20^. Saucerneol F, a new tetrahydrofuran-type sesquilignan isolated from *Saururus chinensis*, has been shown to directly inhibit IKK activity by oxidizing the critical cysteine residue and further inhibiting IκB-α phosphorylation^20^. A similar mechanism of action may be responsible for SGRS inhibiting the phosphorylation of IκB-α and regulating the transcriptional activity of NF-κB. Further, the present study showed that SGRS prevented phosphorylation of p38 and JNK in response to LPS stimulation; however, inhibition of JNK phosphorylation was dominant compared to inhibition of p38 phosphorylation (Fig. 4D). It is evident that SGRS mediated inhibition of MAPKs, leading to transcriptional inactivation of NF-κB, which in turn further down-regulated COX-2 and iNOS expression and suppressed cytokine production. Thus, our findings suggest that SGRS can modulate NF-κB directly via IκB modification or indirectly via MAPK inhibition.

**Figure 6:**
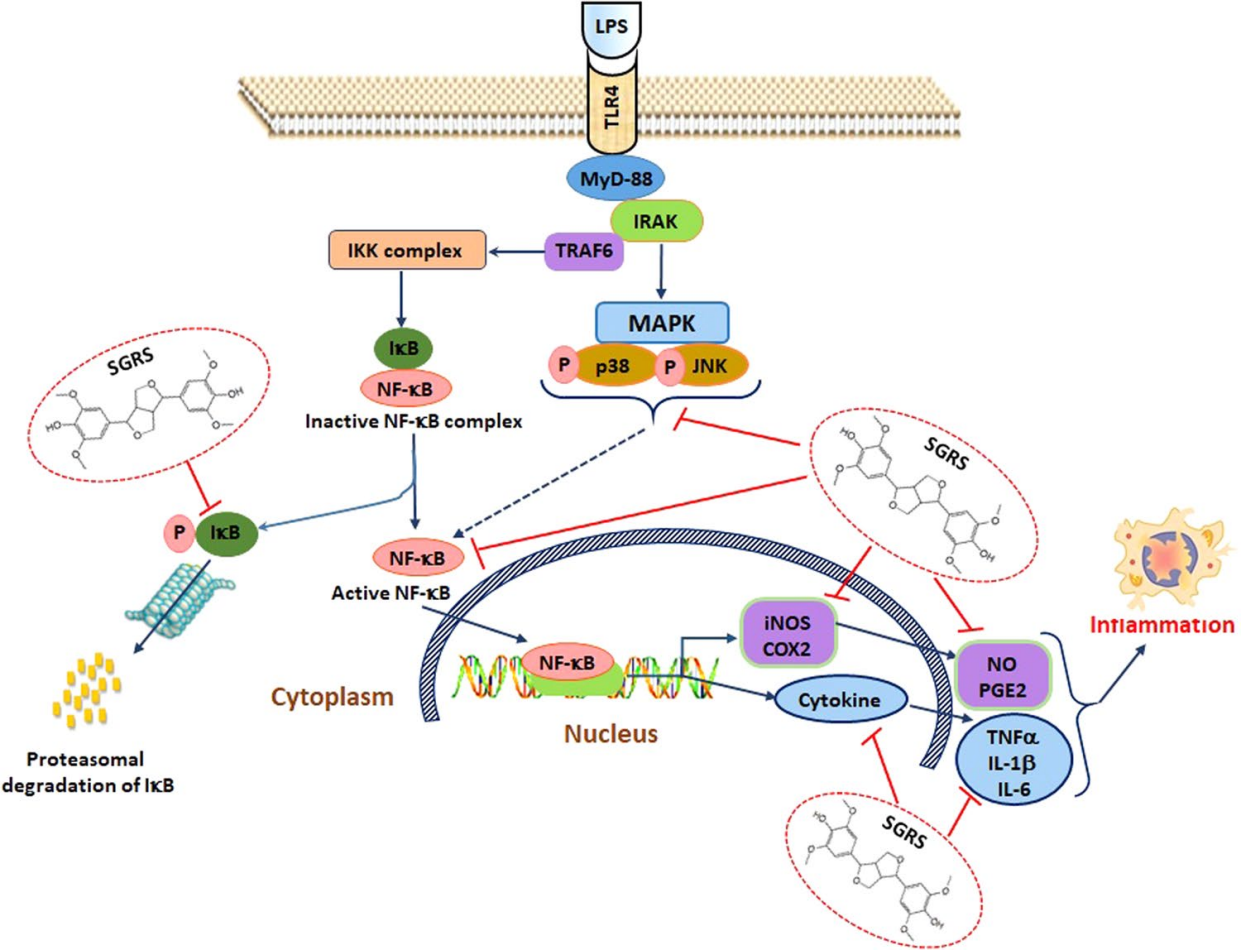
Schematic demonstration of anti-inflammatory mechanistic role of (+)-syringaresinol (SGRS).

Carrageenan as an important phlogistic factor has the ability to induce a variety of inflammatory responses, such as neutrophil-mediated production of free radicals and mediators, neutrophil infiltration, paw edema, and capillary permeability^38,39^. Mounting amount of research has proven carrageenan-induced hind paw acute edematous inflammation assay as an ideal animal model for evaluating the anti-inflammatory potential of any drug molecule^39^. In our study, treatment with SGRS (50 mg/kg/day) resulted in significant reduction of mice paw edema volumes (Fig. 5A). In addition, SGRS treatment also mitigated mRNA expression of iNOS, COX-2, TNF-α, IL-1β, and IL-6 in carrageenan-induced mice compared to the control (Fig. 5B). Subsequently, Western blot analysis revealed that SGRS treatment also suppressed COX-2 as well as NF-κB protein expression in carrageenan-induced mice (Fig. 5C). These findings advocate that the inhibitory mechanism of SGRS against LPS-induced NO, PGE2, TNF-α, IL-1β, and IL-6 production in RAW 264.7 cells may represent an important molecular action resulting in the inhibition of carrageenan-induced formation of paw edema. A systematic mechanism of anti-inflammatory effects of SGRS is summarized in Fig. 6.

This study first time reports isolation and characterization of a lignan, (+)-syringaresinol (SGRS), from *Rubia philippinensis* and demonstrated the anti-inflammatory efficacy of SGRS in LPS-stimulated RAW 264.7 cells *in vitro* and in a carrageenan-induced hind paw edema assay in experimental mice. Collectively, these results conclusively demonstrate that SGRS is an active ingredient of *R. philippinensis* that mediates anti-inflammatory effects by down-regulating NF-κB expression through interference with JNK and p38 phosphorylation and by reducing mRNA levels of iNOS, COX-2, TNF-α, IL-1β, and IL-6, thus suggesting its significant therapeutic potential.

## Electronic supplementary material

Dataset 1

## References

[1] Ran S, Montgomery KE (2012). Macrophage-mediated lymphangiogenesis: the emerging role of macrophages as lymphatic endothelial progenitors. Cancers.

[2] Kamau, J. K., Nthiga, P. M., Mwonjoria, J. K., Ngeranwa, J. J. N. & Ngugi, M. P. Anti-inflammatory activity of methanolic leaf extract of *Kigelia africana* (Lam.) Benth and stem bark extract of *Acacia hockii* De Wild in Mice. *J Dev Drugs* **5**(2) (2016).

[3] Sun LD (2015). Development and mechanism investigation of a new piperlongumine derivative as a potent anti-inflammatory agent. Biochem Pharmacol.

[4] Hiraiwa K, van Eeden SF (2013). Contribution of lung macrophages to the inflammatory responses induced by exposure to air pollutants. Mediators Inflamm.

[5] Chakraborty P, Saraswat G, Kabir SN (2014). Alpha-dihydroxychalcone-glycoside (alpha-DHC) isolated from the heartwood of *Pterocarpus marsupium* inhibits LPS induced MAPK activation and up regulates HO-1 expression in murine RAW 264.7 macrophage. Toxicol Appl Pharmacol.

[6] Lee CW (2014). Biomolecular evidence of anti-inflammatory effects by *Clematis mandshurica* Ruprecht root extract in rodent cells. J Ethnopharmacol.

[7] Lee CW (2015). Hederagenin, a major component of *Clematis mandshurica* Ruprecht root, attenuates inflammatory responses in RAW 264.7 cells and in mice. Int Immunopharmacol.

[8] Ghosh S, Karin M (2002). Missing pieces in the NF-kappaB puzzle. Cell.

[9] Li Q, Verma IM (2002). NF-kappaB regulation in the immune system. Nat Rev Immunol.

[10] Yoshimura A (2006). Signal transduction of inflammatory cytokines and tumor development. Cancer Sci.

[11] Khan S (2012). Anti-inflammatory mechanism of 15,16-epoxy-3alpha-hydroxylabda-8,13(16),14-trien-7-one via inhibition of LPS-induced multicellular signaling pathways. J Nat Prod.

[12] Elmer ADE (1934). Leaflets of Philippine Botany..

[13] Quan KT (2016). Arborinane triterpenoids from *Rubia philippinensis* inhibit proliferation and migration of vascular smooth muscle cells induced by the platelet-derived growth factor. J Nat Prod.

[14] Park HS (2017). Rubiarbonone C inhibits platelet-derived growth factor-induced proliferation and migration of vascular smooth muscle cells through the focal adhesion kinase, MAPK and STAT3 Tyr^705^ signaling pathways. Br J Pharmacol.

[15] Cai XF (2004). Inhibitory lignans against NFAT transcription factor from *Acanthopanax koreanum*. Arch Pharm Res.

[16] Chun K, Alam MB, Son HU, Lee SH (2016). Effect of novel compound LX519290, a derivative of l-allo threonine, on antioxidant potential *in vitro* and *in vivo*. Int J Mol Sci.

[17] Alam MB (2017). Inhibition of melanogenesis by jineol from *Scolopendra subspinipes* mutilans via MAP-Kinase mediated MITF downregulation and the proteasomal degradation of tyrosinase. Sci Rep.

[18] Jung HJ (2003). *In vivo* anti-Inflammatory and antinociceptive effects of liriodendrin isolated from the stem bark of *Acanthopanax senticosus*. Planta Med.

[19] Park JA, Kim HJ, Jin CB, Lee KT, Lee YS (2003). A new pterocarpan, (-)-maackiain sulfate, from the roots of *Sophora subprostrata*. Arch Pharm Res.

[20] Lu Y (2012). Saucerneol F, a new lignan, inhibits iNOS expression via MAPKs, NF-kappaB and AP-1 inactivation in LPS-induced RAW264.7 cells. Int Immunopharmacol.

[21] Axelson M, Sjovall J, Gustafsson BE, Setchell KD (1982). Origin of lignans in mammals and identification of a precursor from plants. Nature.

[22] Korkina L, Kostyuk V, De Luca C, Pastore S (2011). Plant phenylpropanoids as emerging anti-inflammatory agents. Mini Rev Med Chem.

[23] Smeds AI (2007). Quantification of a broad spectrum of lignans in cereals, oilseeds, and nuts. J Agric Food Chem.

[24] Cho YS, Song WS, Yoon SH, Park KY, Kim MH (2018). Syringaresinol suppresses excitatory synaptic transmission and picrotoxin-induced epileptic activity in the hippocampus through presynaptic mechanisms. Neuropharmacol.

[25] Wang S (2017). Syringaresinol-4- O- β- D-glucoside alters lipid and glucose metabolism in HepG2 cells and C2C12 myotubes. Acta Pharm Sin B.

[26] Thach TT, Lee CK, Park HW, Lee SJ, Lee SJ (2016). Syringaresinol induces mitochondrial biogenesis through activation of PPARβ pathway in skeletal muscle cells. Bioorg Med Chem Lett.

[27] Diakos CI, Charles KA, McMillan DC, Clarke SJ (2014). Cancer-related inflammation and treatment effectiveness. Lancet Oncol.

[28] Borthakur A, Bhattacharyya S, Dudeja PK, Tobacman JK (2007). Carrageenan induces interleukin-8 production through distinct Bcl10 pathway in normal human colonic epithelial cells. American journal of physiology. Gastrointes Liver Physiol.

[29] Li C (2016). LFG-500, a newly synthesized flavonoid, attenuates lipopolysaccharide-induced acute lung injury and inflammation in mice. Biochem Pharmacol.

[30] Chen JJ, Wang TY, Hwang TL (2008). Neolignans, a coumarinolignan, lignan derivatives, and a chromene: anti-inflammatory constituents from *Zanthoxylum avicennae*. J Nat Prod.

[31] Kim JY (2009). *In vitro* anti-inflammatory activity of lignans isolated from *Magnolia fargesii*. Bioorg Med Chem Lett.

[32] Zhao F, Wang L, Liu K (2009). *In vitro* anti-inflammatory effects of arctigenin, a lignan from *Arctium lappa* L., through inhibition on iNOS pathway. J Ethnopharmacol.

[33] Wang JP (2011). Topical anti-inflammatory and analgesic activity of kirenol isolated from *Siegesbeckia orientalis*. J Ethnopharmacol.

[34] Corsi L, Zavatti M, Geminiani E, Zanoli P, Baraldi M (2011). Anti-inflammatory activity of the non-peptidyl low molecular weight radical scavenger IAC in carrageenan-induced oedema in rats. J Pharm Pharmacol.

[35] Dhawan P, Richmond A (2002). A novel NF-kappa B-inducing kinase-MAPK signaling pathway up-regulates NF-kappa B activity in melanoma cells. J Biol Chem.

[36] Ci X (2010). Schisantherin A exhibits anti-inflammatory properties by down-regulating NF-kappaB and MAPK signaling pathways in lipopolysaccharide-treated RAW 264.7 cells. Inflammation.

[37] Calixto JB, Campos MM, Otuki MF, Santos AR (2004). Anti-inflammatory compounds of plant origin. Part II. modulation of pro-inflammatory cytokines, chemokines and adhesion molecules. Planta Med.

[38] Handy RL, Moore PK (1998). A comparison of the effects of L-NAME, 7-NI and L-NIL on carrageenan-induced hindpaw oedema and NOS activity. Bri J Pharmacol.

[39] Yang JH (2013). O-Methylated flavonol isorhamnetin prevents acute inflammation through blocking of NF-kappaB activation. Food Chem Toxicol.

