# Supplementary material for: Attenuation of inflammatory responses by (+)-syringaresinol via MAP-Kinase-mediated suppression of NF-κB signaling in vitro and in vivo
Source: Sci Rep. 2018 Jun 15;8:9216. doi: 10.1038/s41598-018-27585-w (PMC6003921; doi:10.1038/s41598-018-27585-w)
Supplement: Supplementary file 1 — Dataset 1 [file 41598_2018_27585_MOESM1_ESM.docx]

**Attenuation of inflammatory responses by (+)-syringaresinol via MAP-Kinase-mediated suppression of NF-κB signaling *in vitro* and *in vivo***

**Vivek K. Bajpai^1^, Md Badrul Alam^2^, Khong Trong Quan^3^, Mi-Kyoung Ju^2^, Rajib Majumder^4,5^, Shruti Shukla^1^, Yun Suk Huh^6^, MinKyun Na^3,*^, Sang Han Lee^2,*^ & Young-Kyu Han^1,*^**

^1^Department of Energy and Materials Engineering, Dongguk University-Seoul, Seoul 04620, Republic of Korea

^2^Departments of Food Science and Biotechnology, Graduate School, Kyungpook National University, Daegu 41566, Republic of Korea

^3^College of Pharmacy, Chungnam National University, Daejeon 34134, Republic of Korea

^4^Department of Biological Sciences, Macquarie University, Sydney, NSW 2109, Australia

^5^Elizabeth Macarthur Agricultural Institute (EMAI), NSW Department of Primary Industries, Menangle, NSW 2567, Australia

^6^Department of Biological Engineering, Inha University, 100 Inha-ro, Nam-gu, Incheon 22212, Republic of Korea

**Running head:** Attenuation of inflammatory responses by a lignan

**Corresponding authors:**

**Dr. Young-Kyu Han;** E-mail: [ykenergy@dongguk.edu](mailto:ykenergy@dongguk.edu); **Dr.** **MinKyun Na;** E-mail: [mkna@cnu.ac.kr](mailto:mkna@cnu.ac.kr); **Dr. Sang Han Lee;** E-mail: [sang@knu.ac.kr](mailto:sang@knu.ac.kr)

**Table S1.** List of the primer sets used in this study.

| Gene name |  | Sequences |
| --- | --- | --- |
| iNOS | forward | GGCTGTCAGAGCCTCGTGGCTTTGG |
|  | reverse | CCCTTCCGAAGTTTCTGGCAGCAGC |
| COX2 | forward | AACACAGCTACGAAAACC |
|  | reverse | CACAGTATGATGTAACAGTCC |
| TNFα | forward | GGCAGGTCTACTTTGGAGTCATTGC |
|  | reverse | ACATTCGAGGCTCCAGTGAATTCGG |
| IL-1β | forward | ATGGCAACTGTTCCTGAACTCAACT |
|  | reverse | CAGGACAGGTATAGATTCTTTCCTT |
| IL6 | forward | GAGGATACCACTCCCAACAGA |
|  | reverse | AAGTGCATCATCGTTGTTCATACA |
| GAPDH | Forward | TTGTGATGGGTGTGAACCAC |
|  | reverse | ACACATTGGGGGTAGGAACA |

**Fig. S1.** ^1^H NMR spectrum of (+)-syringaresinol (300 MHz, MeOD).

**Fig. S2.** ^13^C NMR spectrum of (+)-syringaresinol (75 MHz, MeOD).

**
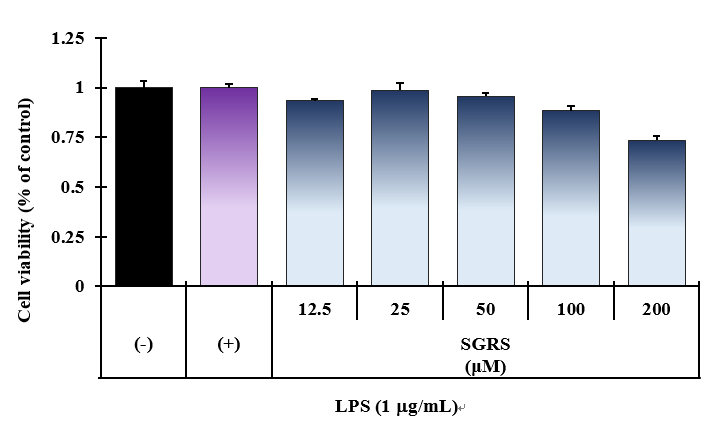
**

**Fig. S3.** Effect of (+)-syringaresinol (SGRS) on the viability of RAW 264.7 cells. Cells were treated with SGRS for 24 h at the indicated concentrations and cell viability was determined by MTT assay. Values are expressed as the mean ± SD (n=3).

**Original gel images**


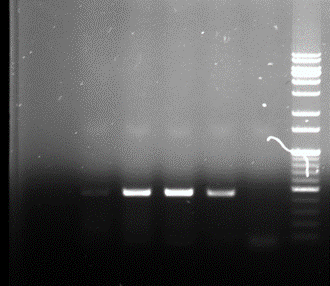


**iNOS**

**
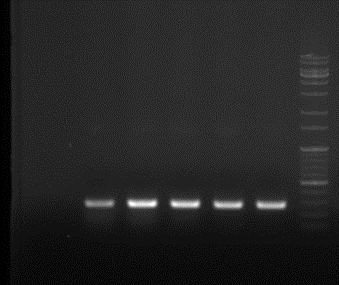
**

**COX-2**

**
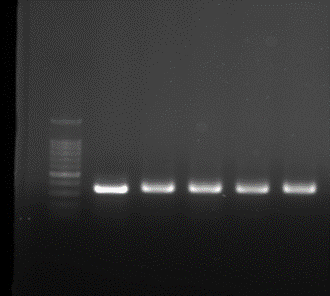
**

**GAPDH**

**Figure 2C (Uncropped original blots)**

***
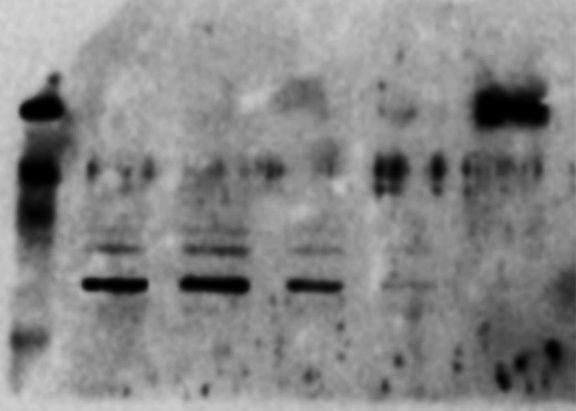
***

**iNOS**

**
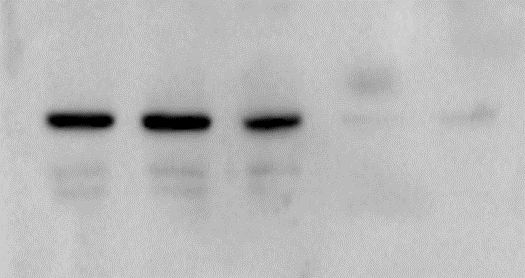
**

**COX-2**


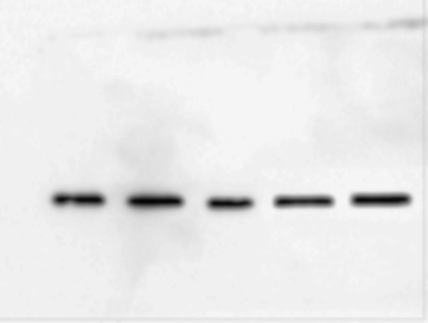


**β-actin**

**Figure 2D (Uncropped original blots)**


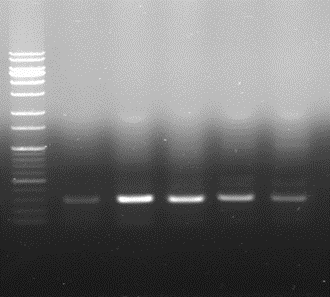


**TNF-α**


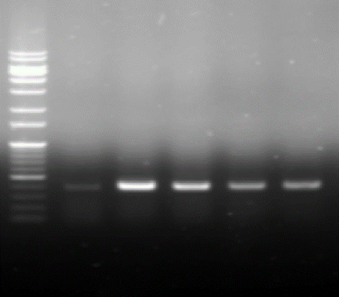


**IL-1β**


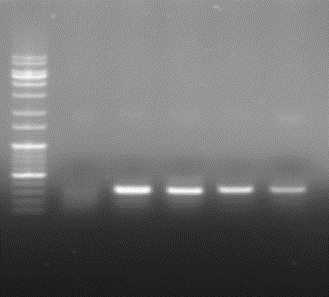


**IL-6**


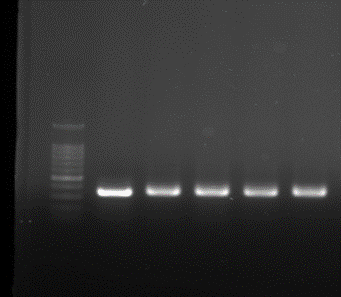


**GAPDH**

**Figure 3D (Uncropped original blots)**


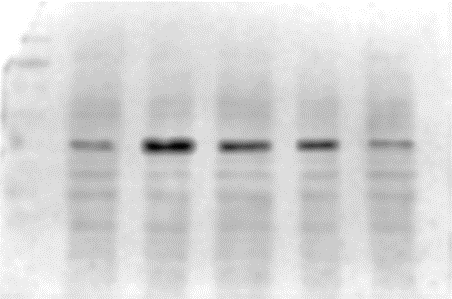


**p-IκB**


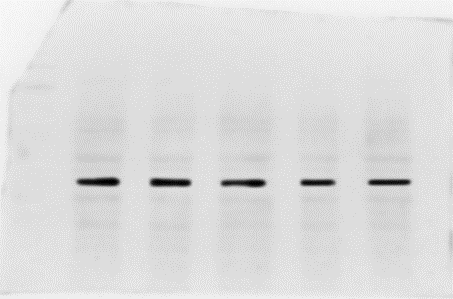


**IκB**


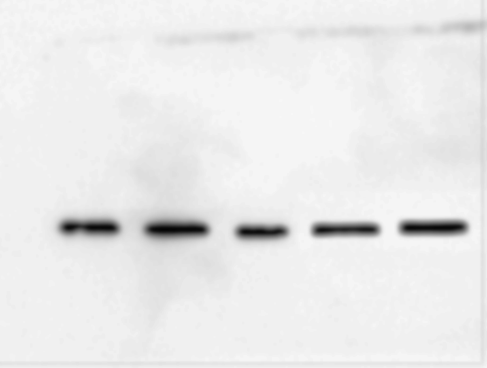


**β-actin**

**Figure 4A (Uncropped original blots)**


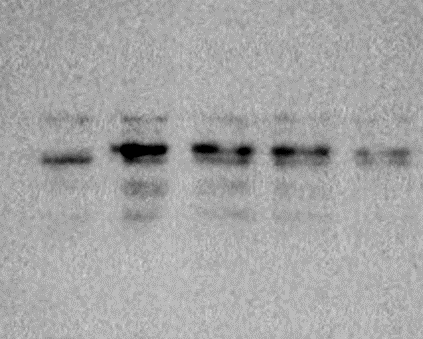


**NF-κB**


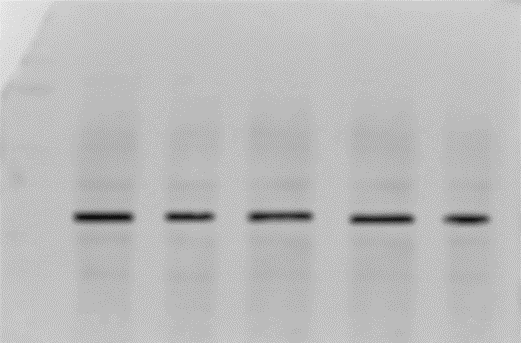


**Lamin B**

**Figure 4B (Uncropped original blots)**


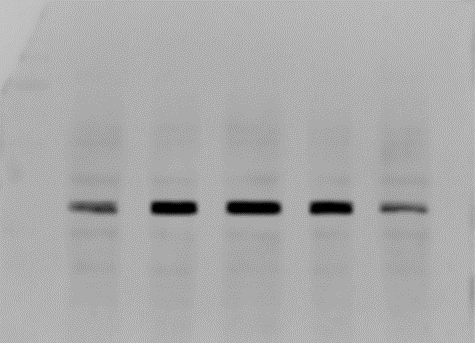


**p-p38**


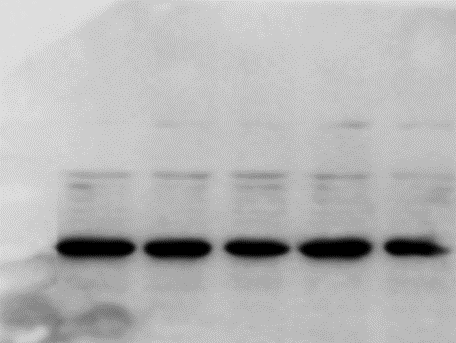


**p38**


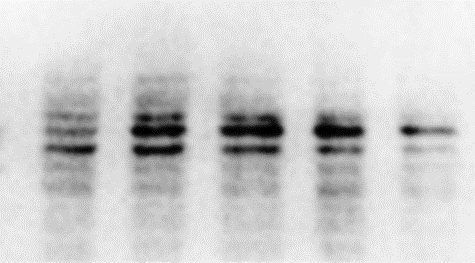


**p-JNK**


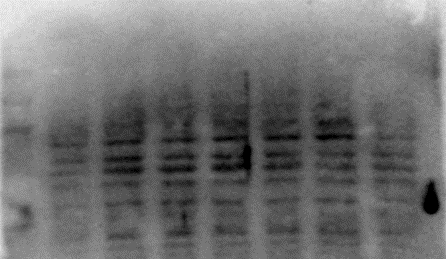


**JNK**


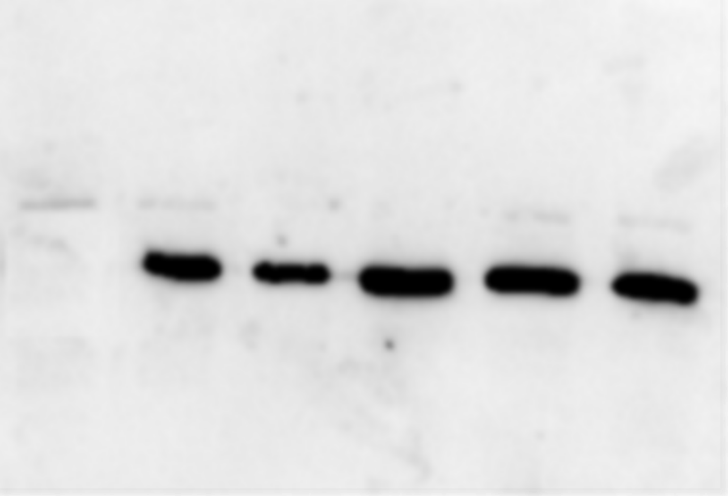


**β-actin**

**Figure 4D (Uncropped original blots)**


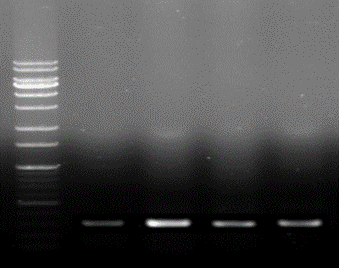


**iNOS**


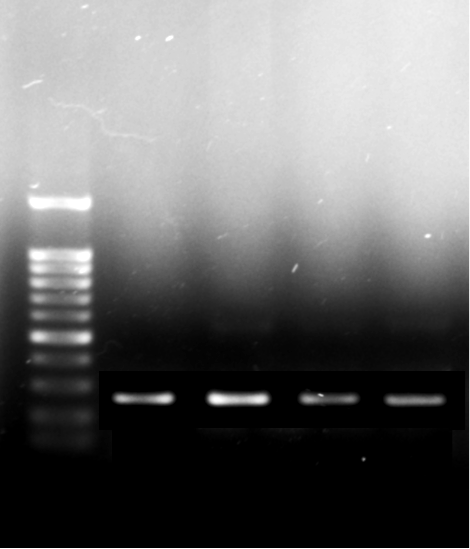


**COX-2**


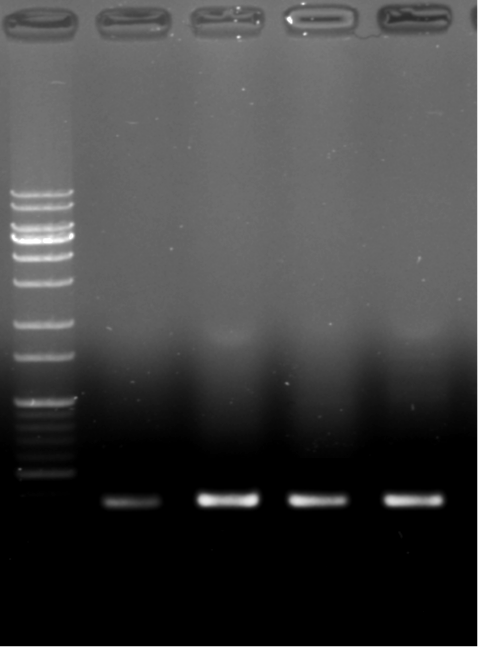


**TNF-α**


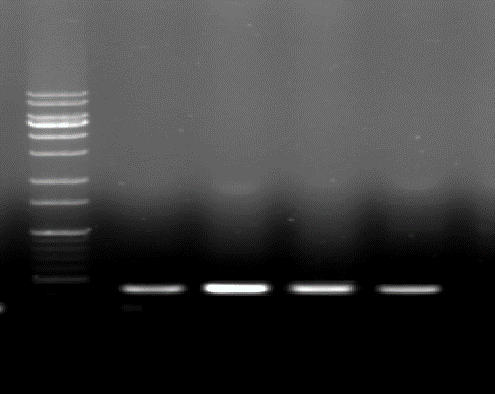


**IL-1β**


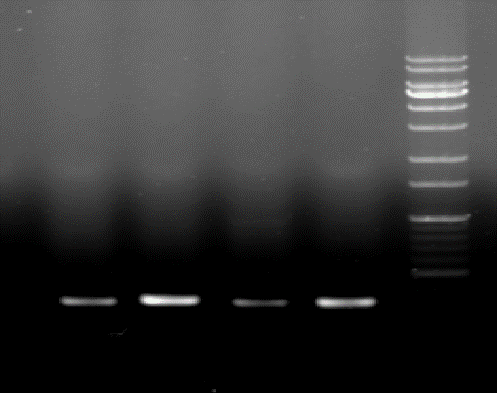


**IL-6**


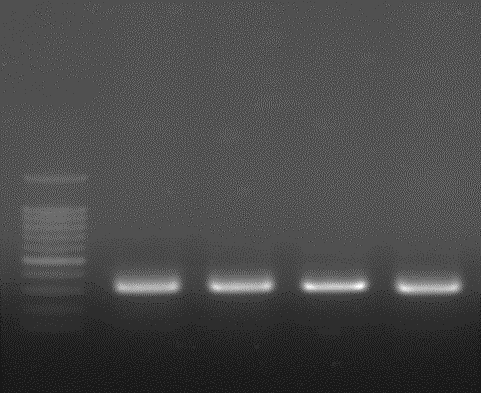


**GAPDH**

**Figure 5B (Uncropped original blots)**


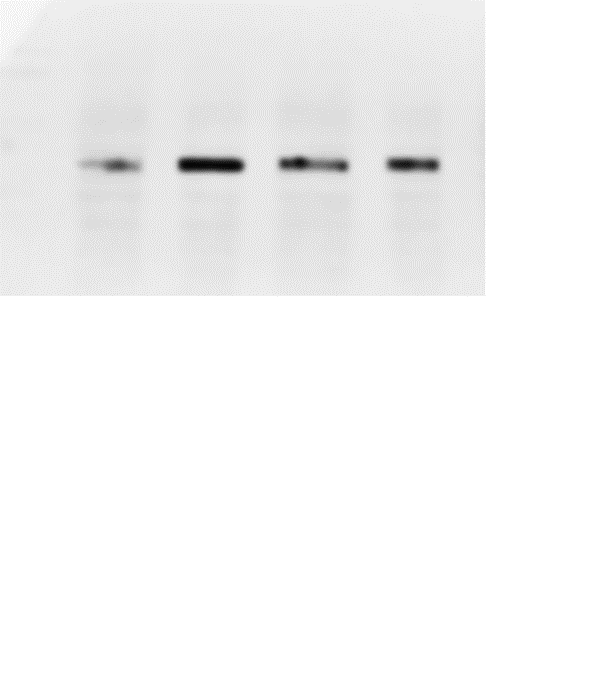


**COX-2**


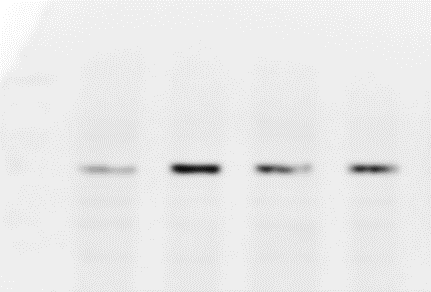


**NF-κB**


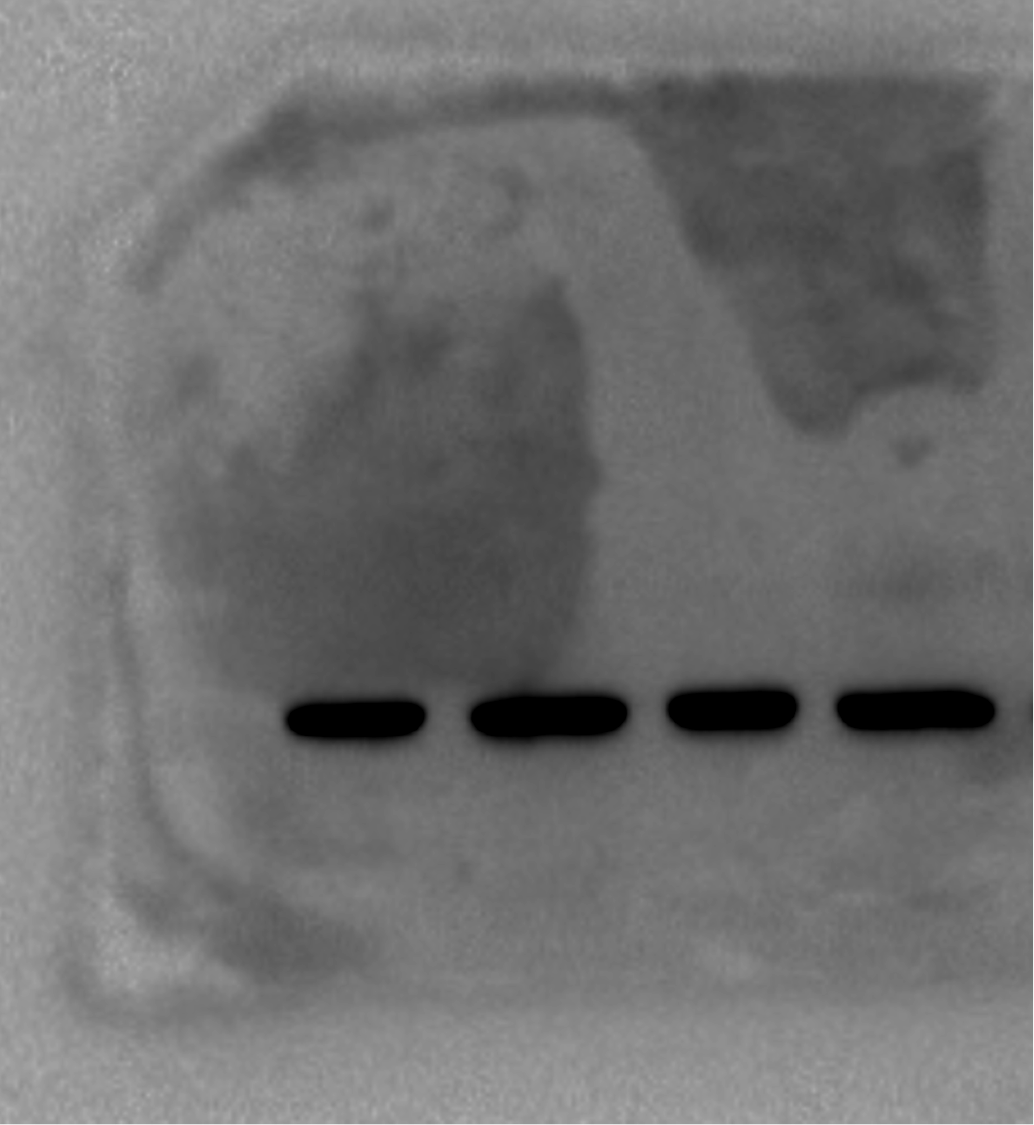


**β-actin**

**Figure 5C (Uncropped original blots)**
